# A deep learning model and human-machine fusion for prediction of EBV-associated gastric cancer from histopathology

**DOI:** 10.1038/s41467-022-30459-5

**Published:** 2022-05-19

**Authors:** Xueyi Zheng, Ruixuan Wang, Xinke Zhang, Yan Sun, Haohuan Zhang, Zihan Zhao, Yuanhang Zheng, Jing Luo, Jiangyu Zhang, Hongmei Wu, Dan Huang, Wenbiao Zhu, Jianning Chen, Qinghua Cao, Hong Zeng, Rongzhen Luo, Peng Li, Lilong Lan, Jingping Yun, Dan Xie, Wei-Shi Zheng, Junhang Luo, Muyan Cai

**Affiliations:** 1 Department of Pathology, Collaborative Innovation Center for Cancer Medicine, State Key Laboratory of Oncology in South China, Sun Yat-sen University Cancer Center, 510060 Guangzhou, China; 2 School of Computer Science and Engineering, Sun Yat-sen University, 510006 Guangzhou, China; 3 Department of Pathology, Tianjin Medical University Cancer Institute and Hospital, 300000 Tianjin, China; 4 Department of Pathology, Affiliated Cancer Hospital & Institute of Guangzhou Medical University, 510095 Guangzhou, China; 5 Department of Pathology, Guangdong Provincial People’s Hospital, Guangdong Academy of Medical Sciences, 510080 Guangzhou, China; 6 Department of Pathology, Fudan University Shanghai Cancer Center, 200032 Shanghai, China; 7 Department of Pathology, Meizhou People’s Hospital, 514011 Meizhou, China; 8 Department of Pathology, The Third Affiliated Hospital, Sun Yat-sen University, 510635 Guangzhou, China; 9 Department of Pathology, The First Affiliated Hospital, Sun Yat-sen University, 510080 Guangzhou, China; 10 Department of Pathology, Sun Yat-Sen Memorial Hospital, Sun Yat-Sen University, 510120 Guangzhou, China; 11 Department of Urology, The First Affiliated Hospital, Sun Yat-Sen University, 510080 Guangzhou, China

**Keywords:** Gastric cancer, Tumour virus infections, Machine learning

## Abstract

Epstein–Barr virus-associated gastric cancer (EBVaGC) shows a robust response to immune checkpoint inhibitors. Therefore, a cost-efficient and accessible tool is needed for discriminating EBV status in patients with gastric cancer. Here we introduce a deep convolutional neural network called EBVNet and its fusion with pathologists for predicting EBVaGC from histopathology. The EBVNet yields an averaged area under the receiver operating curve (AUROC) of 0.969 from the internal cross validation, an AUROC of 0.941 on an external dataset from multiple institutes and an AUROC of 0.895 on The Cancer Genome Atlas dataset. The human-machine fusion significantly improves the diagnostic performance of both the EBVNet and the pathologist. This finding suggests that our EBVNet could provide an innovative approach for the identification of EBVaGC and may help effectively select patients with gastric cancer for immunotherapy.

## Abstract (web summary)

Epstein–Barr virus-associated gastric cancer shows a robust response to immune checkpoint inhibitors. Here the authors introduce a deep convolutional neural network and its fusion with pathologists for predicting it from histopathology.

## Introduction

Gastric cancer (GC) is the fifth most common cancer globally and the fourth leading cause of cancer deaths worldwide^1^. In 2020, there were over one million new cases of GC, with the highest rate of incidence in Eastern Asia^1^. According to The Cancer Genome Atlas (TCGA) Research Network, GCs are classified into four molecular subtypes: Epstein-Barr virus (EBV)-positive tumors, microsatellite instable tumors (MSI), genomically stable tumors, and chromosomal instable tumors^2^. EBV-positive GC, also known as EBV-associated GC (EBVaGC), comprises ~9% of all GC cases and is a distinct subset of gastric cancer^2^ that may respond remarkably well to immune checkpoint inhibitors^3–5^ and have a favorable prognosis^6,7^.

EBV testing is routinely recommended for GC patients in order to identify such a small group of responders for immunotherapy^8^. The most common method for evaluating EBV status in tumor tissues is in situ hybridization (ISH) targeting EBV-encoded small RNAs (EBERs) in histopathologic samples^9^. However, EBV testing by ISH is time-consuming and not cost-saving. Currently, there is no alternative to universal EBV testing. Therefore, a more cost-efficient and accessible tool is needed for confirmatory EBV testing to assist in patient selection, thereby reducing the unnecessary cost for patients with EBV-negative GC (EBVnGC).

Deep learning has been successfully used to identify cancer subtypes and molecular features on hematoxylin and eosin (H&E)-stained histopathological slides, and as such has the potential to serve as a promising cancer biomarker^10,11^. Several studies have demonstrated that deep learning models can accurately predict the MSI status of colorectal cancer through H&E-stained digital whole slide images (WSIs), with an area under the receiver operating curve (AUROC) of 0.77–0.96^12–14^. Moreover, deep learning models can predict the molecular subtype of muscle-invasive bladder cancer from H&E-stained slides^15^ and the hormonal receptor status of breast cancer from histopathological images^16^. Herein, we hypothesize that a deep learning model may facilitate EBVaGC prediction and refine the selection for confirmatory EBV testing.

An image-based deep learning model has the potential to improve visual diagnostic accuracy. In patients with EBVaGC, H&E-stained slides possess some morphological features that could be recognized by pathologists, including poorly differentiated adenocarcinoma and massive lymphocyte infiltration^17,18^. Pathologists triage patients for the confirmative EBV testing on the basis of these features. Besides these recognizable features, a deep learning model might extract more characteristics of EBVaGC that pathologists have not been aware of, consequently predicting EBV status more accurately.

Here we introduce an innovative deep learning model called EBVNet to predict EBV status among patients with GC using H&E-stained slides. More importantly, we further develop a simple yet effective and novel human-machine fusion strategy for the clinical and practical use of the deep learning model.

## Results

### Patients cohorts

Three cohorts were included in this study (Supplementary Fig. 1). The Internal-STAD was used as an internal dataset to develop the EBVNet, enrolling 203 H&E-stained WSIs from 145 patients with EBVaGC and 803 WSIs from 582 patients with EBVnGC in a single medical center. MultiCenter-STAD and TCGA-STAD were served as two independent external validation datasets. MultiCenter-STAD comprised 417 WSIs from 417 patients, including 98 patients with EBVaGC and 319 patients with EBVnGC. TCGA-STAD contained 234 H&E-stained WSIs from 218 patients with EBVnGC and 24 WSIs from 21 patients with EBVaGC. The details of the three datasets were summarized in Supplementary Table 1.

### Diagnostic performances of tumor detector

To fully automate the process of EBV status prediction, a tumor detector was developed based on the internal dataset and used to automatically detect the tumor region of gastric cancer slides on the external datasets. Only the automatically detected tumor regions were used for the prediction of EBV status by the EBVNet. We found that the tumor detector achieved a sensitivity of 0.964 and an AUROC of 0.862 on the MultiCenter-STAD, and a sensitivity of 0.945 and an AUROC of 0.848 on the TCGA-STAD (Supplementary Table 2).

### Performance of EBVNet

**Fig. 1:**
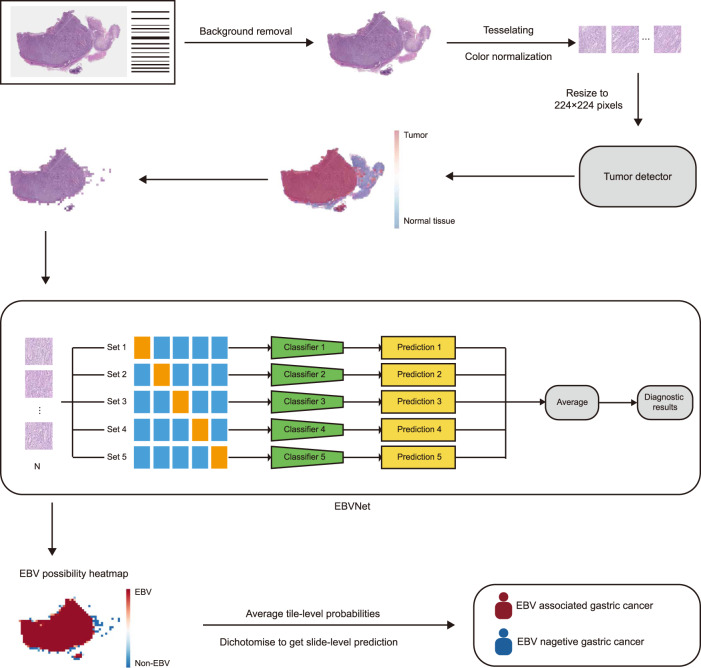
The workflow of EBVNet for predicting EBV status with hematoxylin and eosin-stained WSIs. Each WSI was preprocessed and tessellated into non-overlapped tiles of ×10 magnification. After color normalization, tiles were resized to 224 × 224 pixels and then input to the tumor detector. Only tiles from regions recognized as tumor were fed to EBVNet to get tile-level probabilities for EBV status. The five well-trained individual classifiers were ensembled to form the EBVNet at the output layer of individual classifiers. The average probability outputs of the five individual classifiers were used as the prediction of the ensembled model EBVNet. Tile-level probabilities were averaged to generate a slide-level probability of EBV status. EBV Epstein-Barr Virus, WSI whole slide image.

ResNet50 was utilized as the default backbone for training and validating the EBVNet to predict EBV status of gastric cancer slides (Supplementary Notes and Supplementary Table 3). The workflow of EBVNet was depicted in Fig. 1. On the Internal-STAD, the AUROC of the testing set on each fold ranged from 0.954 to 0.981 (Supplementary Table 4). Over all testing folds, the EBVNet obtained an AUROC of 0.969, a sensitivity of 0.857, specificity of 0.903, and a negative predictive value (NPV) of 0.962.

On the MultiCenter-STAD, EBVNet yielded an AUROC of 0.941 [95% confidence interval (CI) 0.92–0.97], sensitivity of 0.969 (95% CI 0.91–0.99) and specificity of 0.759 (95% CI 0.71–0.81). To mimic the general prevalence of EBVaGC in clinics, we further tested the diagnostic performance 10 times by randomly sampling slides with ~9% proportion being EBVaGC on the MultiCenter-STAD datasets. The AUROC ranged from 0.922 to 0.957 (Supplementary Table 5). The averaged AUROC with ~9% proportion being EBVaGC was similar to that with 23.5% proportion being EBVaGC on the MultiCenter-STAD (0.943 vs 0.941, *P* = 0.720). On the TCGA-STAD, EBVNet achieved an AUROC of 0.895 (95% CI 0.84–0.95), a sensitivity of 0.792 (95% CI 0.58–0.93), and specificity of 0.833 (95% CI 0.78–0.88) (Supplementary Fig. 2). As shown in Supplementary Table 6, the performance of EBVNet was stable for EBV prediction when training with different random seeds.

### Pathologist Reader’s performance

**Fig. 2:**
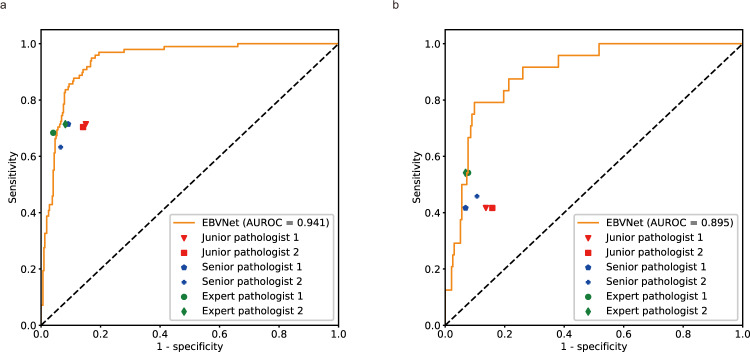
Diagnostic performances of EBVNet and pathologists on two external datasets. **a** On the MultiCenter-STAD, the EBVNet achieved an AUROC of 0.941, outperforming all the six pathologists with an AUROC ranging from 0.782 to 0.821 (*P* < 0.001, Delong’s test, two-sided). **b** On the TCGA-STAD, the EBVNet achieved an AUROC of 0.895, better than all the pathologists with an AUROC ranging from 0.629 to 0.737 (*P* < 0.001, Delong’s test, two-sided). MultiCenter-STAD an external dataset from multiple medical centers, TCGA-STAD an external dataset from The Cancer Genome Atlas, AUROC area under the receiver operating curve.

To compare the diagnostic performance of EBVNet with those of pathologists in predicting EBV status of gastric cancer slides, a pathologist reader study was conducted on MultiCenter-STAD and TCGA-STAD datasets. Pathologists reviewed H&E-stained slides and determined EBV status based on the morphological features. The diagnostic performance improved as the pathologists increased in their years of experience. On the MultiCenter-STAD, Junior pathologist 1, Junior pathologist 2, Senior pathologist 1, Senior pathologist 2, Expert pathologist 1, and Expert pathologist 2 achieved an AUROC of 0.782 (95% CI: 0.74–0.82), 0.782 (95% CI: 0.74–0.82), 0.812 (95% CI: 0.77–0.85), 0.783 (95% CI: 0.74–0.82), 0.821 (95% CI: 0.78–0.86), and 0.816 (95% CI: 0.78–0.85), respectively. Pathologists achieved a sensitivity ranging from 0.633 to 0.714, and specificity from 0.850 to 0.959 on the MultiCenter-STAD. On the TCGA-STAD, Junior pathologist 1, Junior pathologist 2, Senior pathologist 1, Senior pathologist 2, Expert pathologist 1, and Expert pathologist 2 had an AUROC of 0.640 (95% CI: 0.58–0.70), 0.629 (95% CI: 0.57–0.69), 0.674 (95% CI: 0.61–0.73), 0.676 (95% CI: 0.62–0.73), 0.737 (95% CI: 0.68–0.79), and 0.732 (95% CI: 0.67–0.79), respectively. Pathologists yielded a sensitivity from 0.417 to 0.542, and specificity from 0.842 to 0.932. On each of the two external sets, the AUROC of EBVNet was significantly better than that of all the six pathologists (*P* < 0.001) (Fig. 2). The sensitivity of EBVNet was higher than that of all these pathologists, while the specificity of EBVNet was slightly lower than that of pathologists (Table 1). In terms of the interobserver agreement, the Kappa value of junior pathologists, senior pathologists, and expert pathologists was 0.654, 0.719, and 0.698, respectively, on MultiCenter-STAD. On TCGA-STAD, the Kappa value of junior pathologists, senior pathologists, and expert pathologists was 0.552, 0.708, and 0.585, respectively.

**Table 1:** Performance comparison between EBVNet and pathologists on MultiCenter-STAD and TCGA-STAD.

Methods	MultiCenter-STAD						TCGA-STAD					
	Sensitivity	Difference	*P*	Specificity	Difference	*P*	Sensitivity	Difference	*P*	Specificity	Difference	*P*
EBVNet	0.969	NA	NA	0.759	NA	NA	0.792	NA	NA	0.833	NA	NA
Junior pathologist 1	0.714	−0.255	<0.001	0.850	0.091	<0.001	0.417	−0.375	0.004	0.863	0.030	0.337
Junior pathologist 2	0.704	−0.265	<0.001	0.859	0.100	<0.001	0.417	−0.375	0.004	0.842	0.009	0.871
Senior pathologist 1	0.714	−0.255	<0.001	0.909	0.150	<0.001	0.417	−0.375	0.012	0.932	0.099	<0.001
Senior pathologist 2	0.633	−0.336	<0.001	0.934	0.175	<0.001	0.458	−0.334	0.008	0.893	0.060	0.016
Expert pathologist 1	0.684	−0.285	<0.001	0.959	0.200	<0.001	0.542	−0.250	0.070	0.923	0.090	<0.001
Expert pathologist 2	0.714	−0.255	<0.001	0.918	0.159	<0.001	0.542	−0.250	0.070	0.932	0.099	<0.001

Difference indicated the difference between each pathologist and EBVNet. The data have been provided in the Source Data file.

*MultiCenter-STAD* external dataset from multiple medical center, *TCGA-STAD* external dataset from The Cancer Genome Atlas, *NA* not applicable.

### Human-machine fusion

To investigate the application scenario of the deep learning model in clinical practice, we further developed a human-machine fusion strategy to integrate the model into the universal testing paradigm. As shown in Supplementary Table 7, the comparison results of different human-machine fusion strategies indicated that our fusion strategy outperformed other fusion strategies in most cases. Our fusion of the EBVNet and each pathologist with a varying level of expertise further improved the performance of both the EBVNet and the pathologist.

On the MultiCenter-STAD, the prediction fusion from the EBVNet with Junior pathologist 1, Junior pathologist 2, Senior pathologist 1, Senior pathologist 2, Expert pathologist 1, and Expert pathologist 2 achieved an AUROC of 0.945 (95% CI: 0.92–0.97; *P* = 0.581), 0.951 (95% CI: 0.93–0.97; *P* = 0.143), 0.960 (95% CI: 0.94–0.98; *P* = 0.011), 0.960 (95% CI: 0.94–0.98; *P* = 0.008), 0.960 (95% CI: 0.94–0.98; *P* = 0.015), and 0.969 (95% CI: 0.95–0.98; *P* < 0.001), respectively, outperforming that of the EBVNet alone (0.941). The sensitivity of human-machine fusion ranged from 0.878 to 0.969 and specificity ranged from 0.781 to 0.909 on the MultiCenter-STAD.

**Fig. 3:**
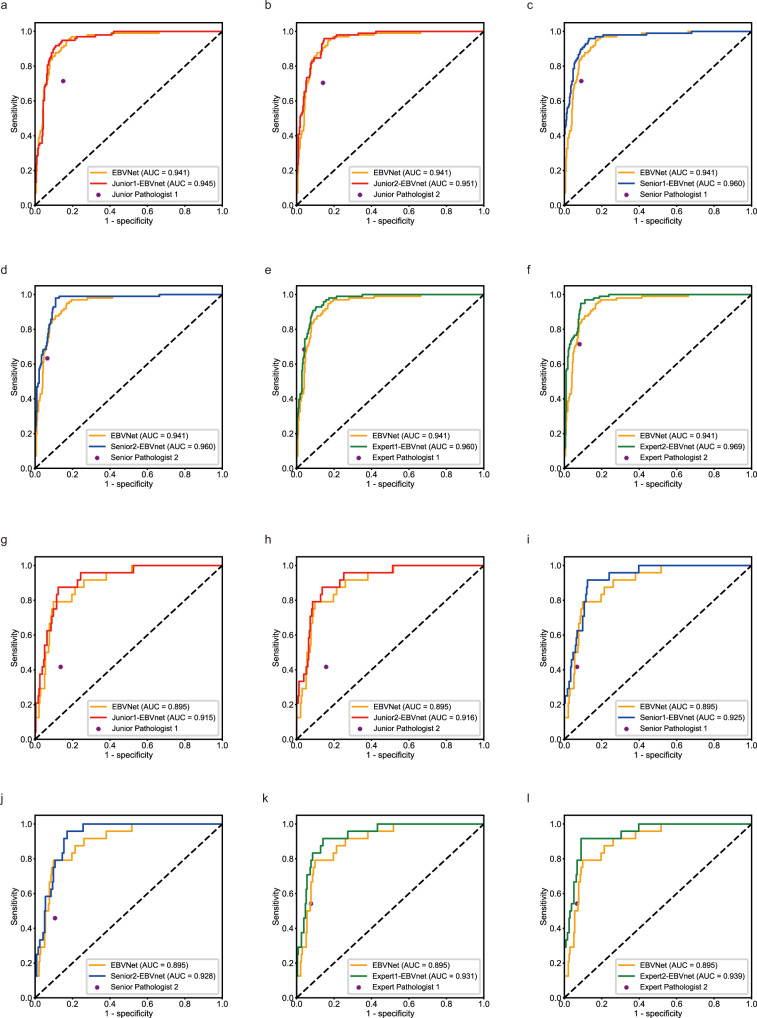
Fusion of predictions from the EBVNet and each pathologist on two external datasets. **a**–**e** On the MultiCenter-STAD, the prediction fusion from the EBVNet and Junior pathologist 1, Junior pathologist 2, Senior pathologist 1, Senior pathologist 2, Expert pathologist 1, and Expert pathologist 2 achieved an AUC of 0.945 (95% CI: 0.92–0.97; *P* = 0.581 when compared with EBVNet by Delong’s test, two-sided), 0.951 (95% CI: 0.93–0.97; *P* = 0.143), 0.960 (95% CI: 0.94–0.98; *P* = 0.011), 0.960 (95% CI: 0.94–0.98; *P* = 0.008), 0.960 (95% CI: 0.94–0.98; *P* = 0.015), and 0.969 (95% CI: 0.95–0.98; *P* < 0.001), respectively, outperforming that of the EBVNet alone (0.941). **g**–**l** On the TCGA-STAD, the prediction fusion from the EBVNet and Junior pathologist 1, Junior pathologist 2, Senior pathologist 1, Senior pathologist 2, Expert pathologist 1, and Expert pathologist 2 yielded an AUC of 0.915 (95% CI: 0.87–0.97; *P* = 0.239), 0.916 (95% CI: 0.87–0.97; *P* = 0.176), 0.925 (95% CI: 0.89–0.97; *P* = 0.167), 0.928 (95% CI: 0.89–0.96; *P* = 0.035), 0.931 (95% CI: 0.89–0.98; *P* = 0.080), and 0.939 (95% CI: 0.90–0.98; *P* < 0.001) respectively, better than that of the EBVNet alone (0.895). MultiCenter-STAD an external dataset from multiple medical centers, TCGA-STAD an external dataset from The Cancer Genome Atlas, AUC area under the receiver operating curve, 95% CI 95% confidence intervals, Junior 1-EBVNet the prediction fusion from the EBVNet and Junior pathologist 1, Junior 2-EBVNet the prediction fusion from the EBVNet and Junior pathologist 2, Senior 1-EBVNet fusion the prediction fusion from the EBVNet and Senior pathologist 1, Senior 2-EBVNet fusion the prediction fusion from the EBVNet and Senior pathologist 2, Expert 1-EBVNet fusion the prediction fusion from the EBVNet and Expert pathologist 1, Expert 2-EBVNet fusion the prediction fusion from the EBVNet and Expert pathologist 2.

On the TCGA-STAD, the prediction fusion from the EBVNet and Junior pathologist 1, Junior pathologist 2, Senior pathologist 1, Senior pathologist 2, Expert pathologist 1, and Expert pathologist 2 yielded an AUROC of 0.915 (95% CI: 0.87–0.97; *P* = 0.239), 0.916 (95% CI: 0.87–0.97; *P* = 0.176), 0.925 (95% CI: 0.89–0.97; *P* = 0.167), 0.928 (95% CI: 0.89–0.96; *P* = 0.035), 0.931 (95% CI: 0.89–0.98; *P* = 0.080), and 0.939 (95% CI: 0.90–0.98; *P* < 0.001), respectively, better than that of the EBVNet alone (0.895) although some results did not reach a significantly statistical difference. The sensitivity of human-machine fusion ranged from 0.625 to 0.917 and specificity ranged from 0.846 to 0.923 on the TCGA-STAD (Table 2 and Fig. 3).

**Table 2:** Prediction fusions from the EBVNet and pathologists with varying levels of expertise.

Method	MultiCenter-STAD			TCGA-STAD		
	Sensitivity	Specificity	AUROC	Sensitivity	Specificity	AUROC
EBVNet	0.969 (0.91, 0.99)	0.759 (0.71, 0.81)	0.941(0.92, 0.97)	0.792 (0.58, 0.93)	0.833(0.78, 0.88)	0.895 (0.84, 0.95)
Junior 1-EBVNet fusion	0.959 (0.90, 0.99)	0.781 (0.73, 0.82)	0.945 (0.92, 0.97)	0.875 (0.68, 0.97)	0.872 (0.82, 0.91)	0.915 (0.87, 0.97)
Junior 2-EBVNet fusion	0.969 (0.91, 0.99)	0.790 (0.74, 0.83)	0.951 (0.93, 0.97)	0.875 (0.68, 0.97)	0.846 (0.79, 0.89)	0.916 (0.87, 0.97)
Senior 1-EBVNet fusion	0.918 (0.85, 0.96)	0.893 (0.85, 0.93)	0.960 (0.94, 0.98)	0.625 (0.41, 0.81)	0.923 (0.88, 0.95)	0.925 (0.89, 0.97)
Senior 2-EBVNet fusion	0.878 (0.80, 0.94)	0.909 (0.87, 0.94)	0.960 (0.94, 0.98)	0.792 (0.58, 0.93)	0.889 (0.84, 0.93)	0.928 (0.89, 0.96)
Expert 1-EBVNet fusion	0.959 (0.90, 0.99)	0.843 (0.80, 0.88)	0.960 (0.94, 0.98)	0.833 (0.63, 0.95)	0.915 (0.87, 0.95)	0.931 (0.89, 0.98)
Expert 1-EBVNet fusion	0.969 (0.91, 0.99)	0.881 (0.84, 0.91)	0.969 (0.95, 0.98)	0.917 (0.73, 0.99)	0.893 (0.85, 0.93)	0.939 (0.90, 0.98)

95% confidence intervals are included in brackets. The data have been provided in the Source Data file.

*Junior 1-EBVNet fusion* the prediction fusion from the EBVNet and Junior pathologist 1, *Junior 2-EBVNet fusion* the prediction fusion from the EBVNet and the Junior pathologist 2, *Senior 1-EBVNet fusion* the prediction fusion from the EBVNet and the Senior pathologist 1, *Senior 2-EBVNet fusion* the prediction fusion from the EBVNet and the Senior pathologist 2, *Expert 1-EBVNet fusion* the prediction fusion from the EBVNet and the Expert pathologist 1, *Expert 2-EBVNet fusion* the prediction fusion from the EBVNet and the Expert pathologist 2, *MultiCenter-STAD* external dataset from multiple medical centers, *TCGA-STAD* external dataset from The Cancer Genome Atlas, *AUROC* area under the receiver operating curve.

### Association between the histopathological features and the EBVaGC prediction

**Fig. 4:**
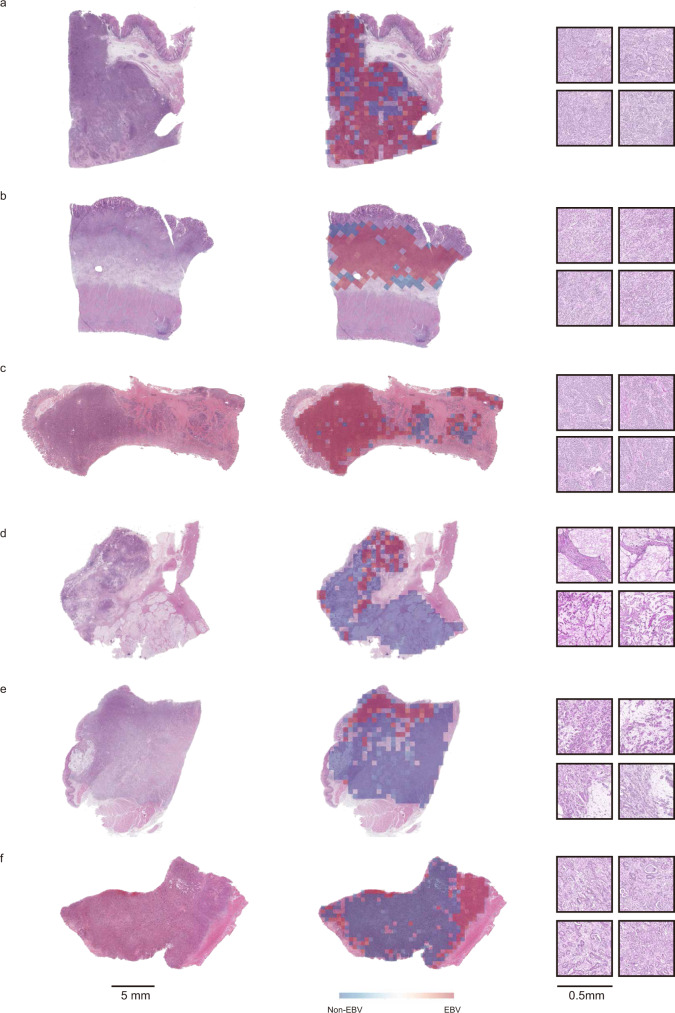
Successful cases predicted by EBVNet. **a**–**c** Histological image (left column) of patients with EBVaGC in **a**–**c** were from Internal-STAD, MultiCenter-STAD, and TCGA-STAD, respectively. The heatmaps overlapped on these three WSIs (middle column) showed that tumor tiles were mainly predicted as EBVaGC with a high score (reddish color). Tiles with a high score were mainly localized in areas of medullary histology, poor differentiation, and tumor with vacuolar nucleus or recognizable nucleolus (right column, tiles at ×10 magnification). **d**–**f** Histological image (left column) of patients with EBVnGC in **d**–**f** were from Internal-STAD, MultiCenter-STAD, and TCGA-STAD, respectively. The heatmaps overlapped on these three WSIs (middle column) showed that tumor tiles were mainly predicted as EBVnGC with a low EBV score (bluish color). All results could be reproduced stably by EBVNet. Tiles with a low score were more likely localized in areas of adenoid differentiation, mucinous differentiation, and signet-ring cell differentiation (right column, tiles at ×10 magnification). EBV Epstein-Barr Virus, EBVaGC Epstein-Barr Virus-associated gastric cancer, EBVnGC Epstein-Barr Virus negative gastric cancer.

To reveal the black-box nature of deep learning model, we further built multivariate logistic regression models to evaluate the association between the histopathological features and the EBVNet’s EBVaGC prediction. On the MultiCenter-STAD, the EBVaGC prediction was significantly correlated with medullary histology [odd ratio (OR), 58.73; *P* < 0.001], mucinous differentiation (OR, 0.30; *P* = 0.011), signet-ring cell differentiation (OR, 0.42; *P* = 0.010), poor differentiation (OR, 5.17; *P* < 0.001). On the TCGA-STAD, the EBVaGC prediction was significantly associated with medullary histology (OR, 9.20; *P* = 0.006), papillary differentiation (OR, 0.17; *P* = 0.003) and vacuolar nucleus or recognizable nucleolus (OR, 3.86; *P* < 0.001) (Fig. 4 and Table 3). The number of morphological features on different datasets are shown in Supplementary Table 8.

**Table 3:** Logistic regression models for the association between the morphological features and EBVNet’s prediction on external datasets.

Features	MultiCenter-STAD				TCGA-STAD			
	Univariate		Multivariate		Univariate		Multivariate	
	OR	*P*	OR	*P*	OR	*P*	OR	*P*
Tertiary lymphoid structure	1.50 (1.00, 2.25)	0.050	NA	NA	1.45 (0.80, 2.61)	0.222	NA	NA
Medullary histology	193.17 (26.49, 1408.74)	<0.001	58.73 (7.75, 445.00)	<0.001	28.60 (6.23, 131.22)	<0.001	9.20 (1.87, 45.22)	0.006
Mucinous differentiation	0.14 (0.06, 0.33)	<0.001	0.30 (0.12, 0.76)	0.011	0.71 (0.32, 1.57)	0.400	NA	NA
Adenoid differentiation	0.41 (0.27, 0.61)	<0.001	0.79 (0.41, 1.53)	0.492	0.43 (0.24, 0.78)	0.005	1.31 (0.53, 3.22)	0.563
Papillary differentiation	0.17 (0.08, 0.36)	<0.001	0.55 (0.22, 1.40)	0.208	0.11 (0.04, 0.33)	<0.001	0.17 (0.05, 0.53)	0.003
Signet-ring cell	0.38 (0.23, 0.63)	<0.001	0.42 (0.22, 0.82)	0.010	0.50 (0.18, 1.34)	0.165	NA	NA
Poor differentiation	7.37 (4.38, 12.40)	<0.001	5.17 (2.46, 10.87)	<0.001	4.02 (2.12, 7.64)	<0.001	2.33 (0.92, 5.90)	0.075
Vacuolar nucleus or recognizable nucleolus	4.30 (2.82, 6.55)	<0.001	1.67 (0.98, 2.84)	0.059	4.11 (2.16, 7.80)	<0.001	3.86 (1.87, 7.98)	<0.001

95% confidence intervals are included in brackets. The data have been provided in the Source Data file.

*OR* odds ratio, *MultiCenter-STAD* external dataset from multiple medical centers, *TCGA-STAD* external dataset from The Cancer Genome Atlas, *NA* not applicable.

### Misdiagnosis from EBVNet

The misdiagnosis of EBVNet was further analyzed to better understand this deep learning model. On the MultiCenter-STAD, the EBVNet misdiagnosed 80 out of 417 slides, including 3 slides of EBVaGC and 77 slides of EBVnGC. Among the 3 misdiagnosed EBVaGC slides, 2 slides were misdiagnosed by all pathologists and the remaining one slide was misdiagnosed by one pathologist. Among the 77 misdiagnosed EBVnGC slides, 7 slides were misdiagnosed by all pathologists, 30 slides were diagnosed correctly by all pathologists, and the remaining 40 slides were misdiagnosed by at least one pathologist. To analyze the morphological features of EBVNet’s misdiagnosed cases, we compared the features of false-positive cases with those of true negative cases and the features of false-negative cases with those of true-positive cases. Compared to true negative cases, these 77 false-positive cases were more likely to occur in female patients (*P* = 0.017), have the presence of medullary histology (*P* < 0.001), poor differentiation (*P* < 0.001), vacuolar nucleus or recognizable nucleolus (*P* = 0.005), and the absence of mucinous differentiation (*P* = 0.008), adenoid differentiation (*P* < 0.001), and papillary differentiation (*P* = 0.008) (Supplementary Fig. 3 and Supplementary Table 9).

On the TCGA -STAD, the EBVNet misdiagnosed 44 out of 258 slides, including 5 EBVaGC slides and 39 EBVnGC slides. Among the 5 misdiagnosed EBVaGC slides, 3 were misdiagnosed by all pathologists and the other 2 were misdiagnosed by at least 1 pathologist. Among the 39 misdiagnosed EBVnGC slides, 4 were misdiagnosed by all pathologists, 15 were diagnosed correctly by all pathologists, and the other 20 were misdiagnosed by at least 1 pathologist. Compared to true negative cases, these 39 false-positive cases were more likely to occur in female patients (*P* = 0.002), have the presence of medullary histology (*P* < 0.001), poor differentiation (*P* = 0.002), and vacuolar nucleus or recognizable nucleolus (*P* < 0.001), and the absence of adenoid differentiation (*P* = 0.036) and papillary differentiation (*P* < 0.001) (Supplementary Table 10).

## Discussion

In this study, we used three diverse datasets to confirm that the innovative deep learning model EBVNet could automatically predict EBV status among gastric cancer H&E-stained WSIs with high performance. Specifically, the EBVNet’s diagnostic performance surpassed that of board-certified pathologists and this model could be generalized to heterogeneous clinical scenarios, including a variety of H&E-stained slides, different medical centers, and patient populations. More importantly, our study further indicated that a human-machine fusion could improve the EBVNet’s performance in identifying EBVaGC, although a further prospective clinical trial would be needed for the confirmation of its validity. These findings suggest that the EBVNet can serve as an efficient approach to identify EBVaGC as well as a promising biomarker to select GC patients for immunotherapy.

To our best knowledge, our study is the first one to report pathologists’ performance in identifying EBV status from H&E-stained slides. Although EBV testing is routinely recommended for GC patients, many GC patients remain EBV-untested due to the high cost and EBV-ISH accessibility. Therefore, only those patients with a high possibility of EBVaGC are selected for EBV testing based on pathologists’ pre-assessments. Although the H&E slides of EBVaGC contain some discriminative features^17,19^, pathologists, even those with more than ten years of specialized gastrointestinal experience, still had poor to moderate interobserver agreements and unsatisfactory diagnostic performances.

Also, this study is the first investigation that compares the performance of a deep learning model to that of pathologists regarding EBVaGC prediction. The AUROC of EBVNet was significantly better than that of all pathologists. Note that the method of calculating the AUROC has been used in dichotomous classification^12,20^, although it might be unfair to pathologists considering that pathologists, in general, cannot give a specific prediction probability score for each data. To draw a fairer comparison, we compared the sensitivity and specificity of EBVNet with those of pathologists according to their dichotomous classification into EBVaGC or EBVnGC^21^. The sensitivity of EBVNet was higher than that of all the pathologists in two external tests while the specificity of EBVNet was slightly lower than that of pathologists. With such a higher sensitivity, the EBVNet can be more potentially served as a screening tool to select patients for the confirmatory EBV testing, thus reducing the misdiagnosis of EBVaGC.

A recent study has shown that a deep learning-based classifier can detect the EBV status with AUROC values ranging from 0.672 to 0.859^22^, but did not report key clinical metrics, such as sensitivity and specificity. More importantly, no comparison was made between the performance of the deep learning model and the human-performed model. Therefore, it remains unknown whether an automated deep learning-based model could provide added value for current clinical EBV testing. In this study, the EBVNet outperformed the published model with AUROCs of 0.941 vs 0.895. A direct comparison between the EBVNet and the published classifier^22^ on the same TCGA database demonstrated that the EBVNet’s performance was superior (AUROC: 0.895 [95% CI: 0.84, 0.95] vs 0.819 [95% CI: 0.73, 0.90]) in detecting EBV status from histopathologic slides, indicating the better effectiveness of our deep learning model in screening patients for the confirmation of EBV status.

It is worth noting that when developing a deep learning model on the Internal-STAD dataset, data imbalance between EBVaGC (minority) and EBVnGC (majority) would cause the developed AI model to predict the majority class (EBVnGC) during inference in external validation or future application. Besides widely used data augmentation techniques for model training, more cases of EBVaGC may directly help the model learn positive features (of the minority class EBVaGC) better. Thus, to develop a EBVNet which can better predict EBVaGC, we included all available slides from the patients with EBVaGC in the Internal-STAD. To analyze the impact of the proportion of EBVaGC on the model performance, we further tested the diagnostic performance by randomly sampling slides with ~9% proportion being EBVaGC on the MultiCenter-STAD dataset. We observed that the EBVNet achieved equivalent AUROCs on the subsets of MultiCenter-STAD and TCGA-STAD (with about 9% prevalence of EBVaGC). Taken together, our results suggest that the diagnostic performance of EBVNet model works very well and is less affected by the proportion of EBVaGC.

Deep learning models have often been regarded as black boxes^10,23^, offering no transparency into how they work. To interpret the EBVNet, we first constructed a logistic regression model to find the features associated with the prediction of the EBVNet. In addition to recognizable features, the EBVNet might be potentially able to extract more characteristics of EBVaGC that have yet to be identified in the previous histopathological study^23^. It is possible that further studies using larger datasets might provide other morphological features that are significantly associated with EBVaGC. By analyzing EBVNet’s misdiagnosed cases, we found that the false-positive cases possessed some morphological features of EBVaGC while the false-negative cases had some characteristics of EBVnGC. Most cases that the EBVNet misdiagnosed were also incorrectly predicted by at least one pathologist, indicating that these cases indeed possessed some confounding features. Taken together, these findings imply that certain effective methods should be developed to overcome this issue and improve the diagnostic performance in future investigations.

Given the prediction uncertainties of both the EBVNet model and the pathologists, we developed and tested a simple yet effective and novel human-machine fusion strategy in these settings. To the best of our knowledge, this is the first study to adaptively fuse predictions from a deep learning model and a human expert based on their prediction uncertainties. To report the prediction confidence of pathologists, we applied the 5-scale self-confidence score method, which is less fine-grained but more clinically practical. The diagnostic performance of the human-machine fusion outperformed that of both the EBVNet and pathologists with varying levels of experience and expertise alone, suggesting that any pathologist could combine the EBVNet’s prediction with his or her own diagnosis to obtain an overall expert-level diagnosis performance. In terms of clinical application, such a human-in-the-loop diagnosis system could be integrated into the current universal testing paradigm in two ways. The first one is to apply the EBVNet as a screening tool. When the prediction of EBVNet encounters a low confidence score, pathologists can help the model perform the prediction. The second way is to let pathologists do the screening based on the morphological characteristics, and EBVNet can assist the pathologists in making the decision when they are not confident enough. The two ways can be potentially applied in the current universal EBV testing paradigm but need further studies to obtain more evidence for the efficacy of the human-in-the-loop system.

While promising results have been obtained from our EBVNet, there do exist several limitations in our study. First, the EBVNet was trained and validated retrospectively, and a rigorous and prospective clinical study is needed to obtain more robust evidence. Second, although the logistic regression model indicated that our EBVNet made biological sense, this method is still an indirect way to interpret the EBVNet. Going forward, more intuitive visualization methods should be attempted in order to interpret the black-box nature of the EBVNet. More importantly, the fusion of the EBVNet and a pathologist should be further evaluated to confirm the improved diagnostic performances in future studies. To potentially further improve the performances of predicting EBV status in gastric cancer slides by deep learning models, besides human-machine fusion, the following aspects may be considered, including the combination of current imaging data with clinical data (tumor manifestation, serum EBV DNA, etc) or multimodality features (like radiomics features), the replacement of network backbones with more recently developed ones (such as Vision Transformer^24^), ensemble model based on different network backbones, and a multi-scale deep learning model by combining different magnifications of slides.

## Methods

### Study participants

This study was approved by the Institutional Review Board of Sun Yat-sen University Cancer Center. To develop EBVNet, we used three pathological image datasets, including the internal dataset from a single medical center (Internal-STAD), the external dataset from multiple medical centers (MultiCenter-STAD), and the well-known public dataset from The Cancer Genome Atlas (TCGA-STAD), to achieve a broad patient representation and improve the ability to generalize our findings. Internal-STAD was served as a training dataset that comprised all available slides from the patients with EBVaGC and randomly-chosen slides from the pool of all patients with EBVnGC in one medical center. On MultiCenter-STAD, the GC patients with available EBV status were randomly included in this study. On TCGA-STAD, patients with the known EBV status were obtained from the TCGA database. The inclusion criteria for this study were the followings: (1) patients with GC underwent primary gastrectomy at the individual hospitals between Jan 1, 2014 and Dec 31, 2020; (2) patients with known EBV status; (3) availability to the clinical data and H&E-stained tumor slides. The exclusion criteria included the followings: (1) patients with preoperative therapy (such as neoadjuvant radiotherapy or chemotherapy); (2) patients with incomplete clinical information; (3) unqualified slide scanning (such as slides out of focus or obvious tissue folds). The informed consent was waived because patients were not directly recruited for this study.

### Slide scanning and annotations

One or two representative H&E-stained tumor slides from each patient’s resection from the Internal-STAD and the MultiCenter-STAD were scanned at ×40 magnification (0.25 μm/pixel) on an Aperio AT2 scanner (Leica Biosystems; Wetzlar, Germany) to generate one or two WSIs in SVS format. The diagnostic slides from TCGA-STAD were downloaded at the Genomic Data Commons portal (https://portal.gdc.cancer.gov/). Blinded to patients’ information and the ground truth of EBV status, two junior pathologists used the software-QuPath opensource^25^ (version 0.2.3) to annotate the slides by drawing regions of interest around the tumor area. Then a senior pathologist checked and revised the annotations. The annotations created by the pathologists were served as the reference standard for tumor detection. Details of WSI processing can be found in Supplementary Methods.

### Determination of EBV status

The ground-truth EBV status from the Internal-STAD and MultiCenter-STAD datasets was determined using ISH targeting EBERs in histopathologic samples at their respective institutions (Supplementary Fig. 4) since EBERs are consistently expressed in all latent EBV infection types^26,27^. The EBV status for the TCGA-STAD was defined by the previously published study through genetic sequencing^2^. Similar sensitivity and reliability between EBV DNA detection and EBER-ISH were observed in the previous study, suggesting that EBERs ISH was interchangeable with genetic sequencing for identifying EBV status^28^.

### Tumor detector

To fully automate the analysis of gastric cancer WSIs, a tumor detector was developed mainly based on the internal dataset and then used to automatically find the tumor regions in each slide from the two external validation datasets. The detected tumor regions were then further analyzed by the EBVNet. A well-known convolutional neural network ResNet50 was used as the classifier backbone for the tumor detector. For tumor detection, 1006 GC slides of Internal-STAD manually outlined by pathologists were set as tumor tissues. In GC cases with diffuse type, it is very difficult to define clear boundaries between adjacent normal mucus and tumor. Thus, we randomly selected 145 additional gastric tissues free of tumors and set them as normal tissues. In this study, the size of each tile is 512-by-512 pixels with a magnification ×10. During training, the dataset was split into five-folds, and the five-fold cross-validation strategy was adopted to train five individual tumor classifiers, each time using four folds to train the classifier and another fold of data as internal validation set to determine when to stop the training (i.e., when the performance of the classifier does not further improve on the internal validation set). Stochastic gradient descent (SGD) optimizer with batch size 64 and weight decay 0.0005 was used to train each classifier for maximally 50 epochs. The learning rate starts from 0.001 and changes with a cosine annealing schedule. After five individual tumor classifiers were well trained, for any input tile from a new slide on the external datasets, the probability outputs of the five classifiers were averaged as the final output of the ensemble tumor detector. The input tile was classified as ‘tumor’ class when the average probability is larger than 0.5.

### EBVNet development

To predict whether a patient belongs to the EBV subgroup or not, an ensemble binary classifier called EBVNet with any network backbone (e.g., ResNet50, VGGNet, EfficientNet) can be trained based on the internal dataset from Internal-STAD. During training, the dataset was divided into five-folds at the slide level, and the five-fold cross-validation strategy was employed to train five individual classifiers first. In particular, for each individual classifier, four folds of data were used to train the classifier and the remaining one was utilized as an internal validation set to determine when to stop training the classifier. For the training of each classifier, each slide was regularly divided into multiple tiles (i.e., image patches) with tile sizes 512-by-512 pixels, and only the tiles from the tumor regions and their labels (1 for ‘EBV’, 0 for ‘non-EBV’) were used respectively as the inputs and the expected outputs of the classifier. A tile in a slide is considered from the tumor region when 50% pixels of the tile are within the pre-segmented tumor region in the slide. About 52,600 EBV tiles and 178,500 non-EBV tiles were obtained for the training of each individual classifier, and about 15,600 EBV tiles and 63,400 non-EBV tiles for internal validation. For each individual classifier, SGD optimizer with batch size 64 and weight decay 0.0005 was used to train the model for maximally 150 epochs. The learning rate starts from 0.001 and changes with a cosine annealing schedule. The training was stopped when the classifier performance on the internal validation set was not further improved over 5 consecutive epochs or at the last (maximum) epoch. It has been consistently observed that classifier training converged after 100 epochs or so. Such classifier training process was repeated five times to generate five individual classifiers, each time with a different fold as the internal validation set. The five well-trained individual classifiers were ensembled to form the EBVNet at the output layer of individual classifiers, i.e., the average probability outputs of the five individual classifiers are used as the prediction of the ensembled model EBVNet. Since the ensemble EBVNet was used to predict EBV status for each tile rather than for each slide, to predict the EBV status of any slide, the EBVNet predictions over all tiles from the tumor regions in the slide were averaged as the final EBV prediction probability for the slide. The diagnostic performances of different model backbones (including VGGNet16, ResNet18, ResNet50, SE_ResNet50, DenseNet121, EfficientNet-B0 and EfficientNet-B1) on Internal-STAD were compared.

### EBVNet evaluation

The EBVNet was internally evaluated with the Internal-STAD dataset and externally assessed with two external datasets, the MultiCenter-STAD and TCGA-STAD. For the external testing, the developed ensembled EBVNet classifier was used to predict the EBV probability at the slide level, and the predictions were compared with the ground-truth EBV status for each external dataset. For the internal evaluation, to faithfully simulate the external evaluation scenario, the five-fold cross-validation strategy was applied as follows. First, one-fold of the internal dataset was held-out as the simulated external test set, and the other four folds were further divided into five new subsets to train an ensemble AI model for the external evaluation. Then, the ensemble model was evaluated on the held-out one-fold at the slide level. Such a process was repeated five times, each time with a different one-fold as the simulated test set. In this way, every slide on the internal dataset was used once for evaluation, and the EBV status predictions of all slides were finally compared with the corresponding ground-truth EBV status. Only tiles from tumor regions were used to train and evaluate the model for both internal and external evaluations.

### Pathologist reader study

To investigate whether the EBV status could be identified by pathologists from H&E-stained slides alone, six pathologists with different years of experience (two junior pathologists with less than 5 years of experience; two senior pathologists with about 10 years of experience; and two experts with specialized in gastric cancer with up to 15 years of experience) were presented with slides from the MultiCenter-STAD and TCGA-STAD. Blind to all clinical information and the performance of EBVNet, pathologists reviewed these slides and classified each case into EBVaGC or EBVnGC based on their expertise and experience.

To obtain further insight into associations between specific histopathological features and EBVNet’s predictions (i.e., EBVaGC or EBVnGC), a logistic regression model was built to assess the relationship between the histopathological features and the EBVNet’s prediction. Based on previous studies^29^, EBVaGC is associated with some morphological features, including poorly differentiation and tertiary lymphoid structure. All these reported morphological features are positive features of EBVaGC but no negative feature reported. Based on the pathologist’s expert experience with gastric cancer, EBVaGC is also linked to some other positive morphological features like medullary histology and vacuolar nucleus (most chromatin distributed at the peripheral nucleus rather than the central nucleus) or recognizable nucleolus, and negative features like mucinous differentiation, adenoid differentiation (tumor cells arranged in glandular patterns), papillary differentiation and signet-ring cell. Therefore, these eight histopathological features were all included in this study. Two expert pathologists worked together to determine whether each of the features was present in individual cases.

To further understand EBVNet, we assessed the association between morphological features and the misdiagnosis of EBVNet. The morphological features of false-negative cases were compared with that of true-positive cases while the features of false-positive cases were compared with that of true negative cases.

### Human-machine fusion

EBVNet can not only be used to predict EBV status of patients in a standalone manner, but also be combined with pathologist predictions in a human-machine fusion manner. In this study, we proposed a simple yet novel adaptive fusion strategy to combine the predictions of the deep learning model EBVNet and pathologists with varying degrees of experience, mainly based on their prediction uncertainties. The details of the human-machine fusion are described below, with the overall fusion strategy introduced first, followed by the design of prediction uncertainty for both EBVNet and pathologists. It is worth noting that the human-machine fusion is predefined and not involved in the training of EBVNet.

Suppose an EBVNet has been well trained and prepared to collaborate with a pathologist to predict the EBV status of a patient. Based on the slide data of the patient, denote by **P**_*m*_ and **P**_*h*_ the two-dimensional output probability vectors from the EBVNet and the pathologist, respectively, and *u*_*m*_ and *u*_*h*_ the prediction uncertainties from the EBVNet and the pathologist, respectively. Then, $\frac{1}{{u}_{m}}$ and $\frac{1}{{u}_{h}}$ can represent prediction certainties (or confidence) from the EBVNet and the pathologist, respectively. Based on the EBV predictions and prediction certainties from both the EBVNet and the pathologist, the output of the human-machine fusion, i.e., the fused prediction **P**_*f*_ from the EBVNet and the pathologist, can be defined as

1 $${{{{{{\bf{p}}}}}}}_{f}=\alpha \cdot {{{{{{\bf{p}}}}}}}_{m}+\left(1-\alpha \right)\cdot {{{{{{\bf{p}}}}}}}_{h},$$

where

$$\alpha =\frac{\frac{1}{{u}_{m}}}{\frac{1}{{u}_{m}}+\frac{1}{{u}_{h}}}$$

Here *α* represents the relative importance of the prediction from the EBVNet for the final fusion prediction **P**_*f*_, and similarly 1 − *α* represents the relative importance of the prediction from the pathologist. Intuitively, when the EBVNet is more certain than the pathologist for the EBV prediction, the final prediction **P**_*f*_ will be more dependent on the model prediction **P**_*m*_, and vice versa. From Eq. (1), the main challenge is to obtain the pathologist’s prediction probability **P**_*h*_ and the two prediction uncertainties *u*_*m*_ and *u*_*h*_.

For the uncertainty *u*_*m*_ of the EBVNet prediction, a deep learning model may be uncertain under two conditions, either when the new slide data are very different from all those used for model training (called knowledge uncertainty), or when the new slide data is similar to one or more slides from both the EBV and non-EBV classes used for model training (called data uncertainty). Fortunately, when the deep learning model is an ensemble of multiple individual models (as EBVNet), both types of prediction uncertainties can be captured by the entropy of the ensemble model’s probability output **P**_*m*_^21,30^. Therefore, the uncertainty *u*_*m*_ for EBVNet prediction can be estimated by.

2 $${u}_{m}=-\left({p}_{m,1}\cdot {{{{{\rm{ln}}}}}}{p}_{m,1}+{p}_{m,2}\cdot {{{{{\rm{ln}}}}}}{p}_{m,2}\right),$$

where *p*_*m*,1_ and *p*_*m*,2_ are respectively the first and second component of the probability output **P**_*m*_ of the EBVNet.

In order to obtain the pathologist’s prediction probability **P**_*h*_ and the associated uncertainty *u*_*h*_, we collected not only his or her diagnosis result (‘EBV’ or ‘Non-EBV’), but also his or her 5-scale self-confidence on the diagnosis, with ‘1’ to ‘5’, respectively, representing ‘surely non-EBV’, ‘likely non-EBV’, ‘unsure’, ‘likely EBV’, and ‘surely EBV’. The five self-confidence scales [1, 2, 3, 4, 5] were simply linearly transformed to the corresponding probabilities [0.2, 0.35, 0.5, 0.65, 0.8], where the smallest and largest probability are respectively set to 0.2 and 0.8 (rather than 0 and 1) by considering the potential over-confidence or noise in reported self-confidence. With this transformed probability, a two-dimensional probability output **P**_*h*_ can easily be obtained based on the pathologist’s diagnostic result and his or her self-confidence report. For example, for the diagnosis result ‘EBV’ and self-confidence ‘5’, **P**_*h*_ would be [0.8, 0.2] where the first component represents the probability of being ‘EBV’; and for the diagnosis result ‘non-EBV’ and self-confidence ‘2’, **P**_*h*_ would be [0.35, 0.65]. Once **P**_*h*_ is obtained, the associated prediction uncertainty *u*_*h*_ can be easily estimated by the entropy of **P**_*h*_, similarly based on Eq. (2). Finally, after obtaining the pathologist’s prediction **P**_*h*_ and prediction uncertainty *u*_*h*_, together with the EBVNet prediction **P**_*m*_ and prediction uncertainty *u*_*m*_, the human-machine fusion output can be obtained based on the designed fusion strategy (Eq. 1).

Besides the proposed human-machine fusion strategy, other human-machine fusion strategies (‘Or’ strategy, ‘And’ strategy, and ‘1-uncertainty’ strategy) have been described in the Supplementary Methods. The diagnostic performances of different human-machine fusion strategies on two external datasets were compared.

### Statistical analysis

Using the ground-truth EBV status as the reference standard, the AUROCs of EBVNet were calculated according to its prediction scores and the AUROCs of pathologists were determined based on their dichotomous classification into EBVaGC or EBVnGC^20^. Therefore, in the ROC space, each pathologist corresponds to a point and EBVNet can provide a continuous curve^12^. The AUROC of each pathologist was calculated by the area under two lines that link the pathologist’s point to (0,0) and (1,1) in the axis, respectively. The AUROC were calculated and compared by Delong’s test. The cutoff threshold of EBVNet’s receiver operator characteristic curve was defined by Youden’s *J* statistic^31^ to dichotomize EBVNet’s probabilities into binary predictions for calculating the sensitivity, specificity, and NPV. This threshold was predefined and determined by the Internal-STAD before the evaluation of the external datasets. The sensitivity and specificity were compared using the McNemar test. The baseline data of study participants from different datasets were compared by variance analysis or Chi-square test. The morphological features of misdiagnosed cases were compared with those of correctly diagnosed cases with the Chi-square test or *t-*test. In terms of the interobserver agreement, the Kappa value of different-level pathologists was calculated with the Chi-square test. The associations between EBVNet prediction and morphological features were obtained by logistic regression models. The 95% CIs of the AUROC were calculated by bootstrapping. Differences were considered significant when the *P-*value from a two-tailed test was less than 0.05. IBM SPSS Statistics (version 20.0) and Medcalc (vesion 15.2.2) were used for statistical analysis. Python (version 3.9.6) and the deep learning platform PyTroch (version 1.9) were used to build the model and analyze the data.

### Reporting summary

Further information on research design is available in the Nature Research Reporting Summary linked to this article.

## Supplementary information

Supplementary Information

Reporting Summary

## Data Availability

The TCGA diagnostic whole slides and corresponding labels are available from NIH genomic data commons (https://portal.gdc.cancer.gov/). Restrictions are applied to the whole slide images and annotation data of Internal-STAD and MultiCenter-STAD, which are used with institutional permission via IRB approval for the current study, and thus are not publicly available due to patient privacy obligations. All data supporting the findings of this study are available on requests for non-commercial and academic purposes from the corresponding author M.C. (caimy@sysucc.org.cn) within 10 working days. We do not require to sign a data use agreement. Processed data can be reproduced stably by the source code. Source data are provided as a zip file with the paper. Source data are provided with this paper.

## Source data

Source Data

## Code availability

Source code can be available at https://github.com/Kepler1647b/EBVnet.
