# Supplementary material for: A deep learning model and human-machine fusion for prediction of EBV-associated gastric cancer from histopathology
Source: Nat Commun. 2022 May 19;13:2790. doi: 10.1038/s41467-022-30459-5 (PMC9120175; doi:10.1038/s41467-022-30459-5)
Supplement: Supplementary file 1 — Supplementary Information [file 41467_2022_30459_MOESM1_ESM.pdf]

A deep learning model and human-machine fusion for prediction of EBV-associated gastric cancer from histopathology

Supplementary Information

Supplementary Methods

WSI preprocessing

Each slide contained tissue regions and blank background regions. Since only tissue regions were informative, tissue regions of each slide were extracted simply by first transforming the down-sampled slide (at 10× magnification) from the original red-green-blue (RGB) to the gray version, and then thresholding the gray image with an experimentally determined threshold value (i.e., 220). 10× magnification has been chosen in this study because some features associated with EBV-associated gastric cancer (EBVaGC) might be better recognized at a lower magnification. The segmented tissue region mask in the down-sampled slide was then up-sampled to obtain the corresponding tissue regions from the original slide. Color normalization[1] on the tissue regions was also performed to reduce potential color variations due to the difference in histological staining between healthcare centers. Additionally, for each slide from the internal cohort (Internal-STAD), tumor regions were manually annotated for the development of an automatic tumor region detector and EBVNet in the next step. Each slide of magnification at 10× was tiled into non-overlapping patches 512×512 pixels in size. The tiles from the Internal-STAD with more than 50 percent region from tumor tissue were marked as tumor tiles, and the rest were marked as normal tiles.

Different human-machine fusion strategies

For human-machine fusion, the following fusion strategies have been applied in medical image classification:

(1) 'Or' strategy: When human experts can only provide diagnosis results (without confidence scores), a patient is diagnosed positive if either the human expert or the AI model considers the patient to be positive based on analysis of the

patient's imaging data[2].

(2) 'And' strategy: When human experts can only provide diagnosis results (without confidence scores), a patient is diagnosed positive if both the human expert and the AI model considers the patient to be positive based on analysis of the patient's imaging data.

(3) '1-uncertainty' strategy: When human experts can provide both diagnosis results and confidence information somehow (e.g., as used in our study), the relative importance α of the prediction for EBVnet was alternatively defined as $\frac{1-u_m}{1-u_m+1-u_h}$ for the final fusion prediction $\mathbf{p}_f$. Note that such alternative calculation of α is for our fusion strategy (Equation 1 in the main manuscript), and the adopted definition (i.e., $\alpha = \frac{\frac{1}{u_m}}{\frac{1}{u_m}+\frac{1}{u_h}}$) in the main manuscript is named as '1/uncertainty' strategy in Supplementary Table 7.

Supplementary Notes

Diagnostic performances of different models for predicting EBV status on Internal-STAD

The diagnostic performances of empirical models with different model backbones (including VGGNet16, ResNet18, ResNet50, SE_ResNet50, DenseNet121, EfficientNet-B0 and EfficientNet-B1) were further evaluated on the internal dataset. The results demonstrated that similar classification performances were obtained by the proposed ensemble AI models (Supplementary Table 3). Among these model backbones, ResNet50 achieved the second highest AUROC of 0.969, but with far less parameters than VGGNet16 which yielded the highest AUROC. Thus, EBVNet with ResNet50 was utilized as the default backbone for further investigations.

Supplementary Figures

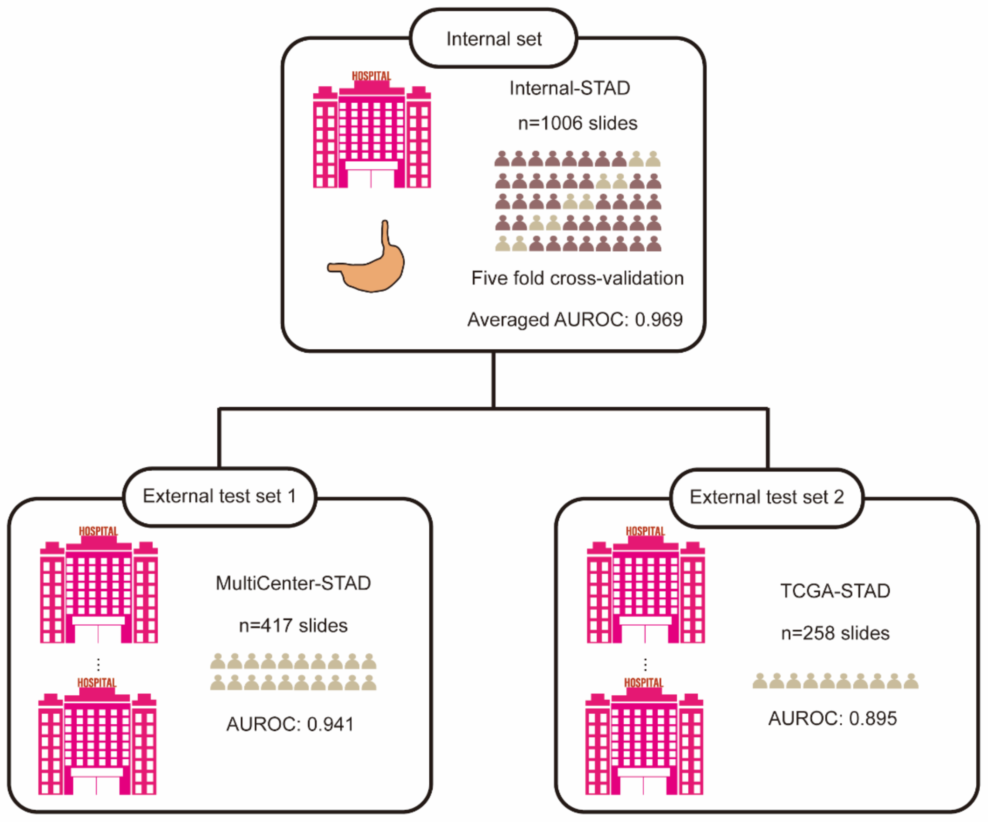

Supplementary Fig. 1. **Brief usage summary of the internal set and the two external sets.** A deep learning model was first developed and internally validated using 1006 slides from a single medical center (Internal-STAD), achieving an AUROC of 0.969. The developed EBVNet was then validated externally on the multicenter dataset of 417 slides and on the TCGA dataset of 258 slides, yielding an AUROC of 0.941 and 0.895, respectively. MultiCenter-STAD, an external dataset from multiple medical centers; TCGA-STAD, an external dataset from The Cancer Genome Atlas; AUROC, area under the receiver operating curve.

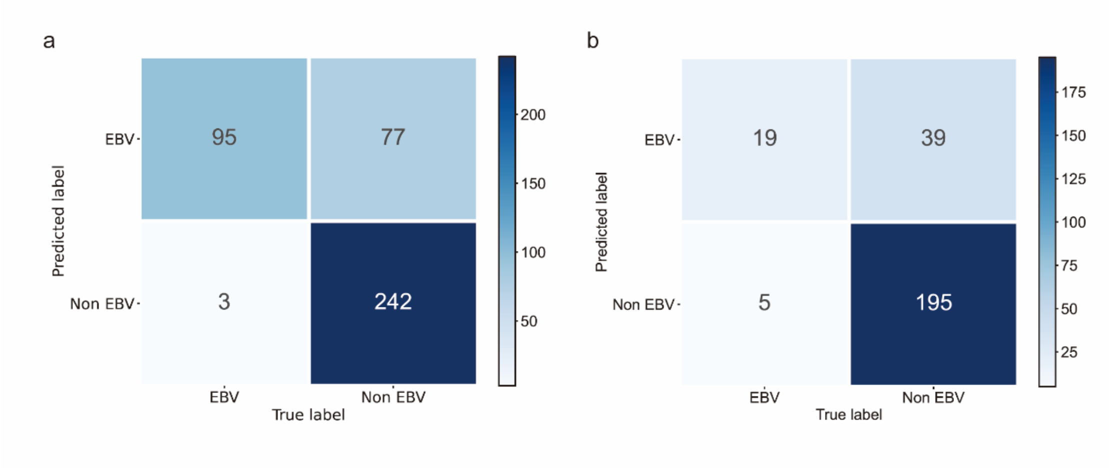

Supplementary Fig. 2. **Confusion matrix based on the ground-truth EBV status of all patients and predictions by EBVNet. a** The confusion matrix for the MultiCenter-STAD. **b** The confusion matrix for the TCGA-STAD. Frequencies are displayed on a color gradient scale. MultiCenter-STAD, an external dataset from multiple medical centers; TCGA-STAD, an external dataset from The Cancer Genome Atlas.

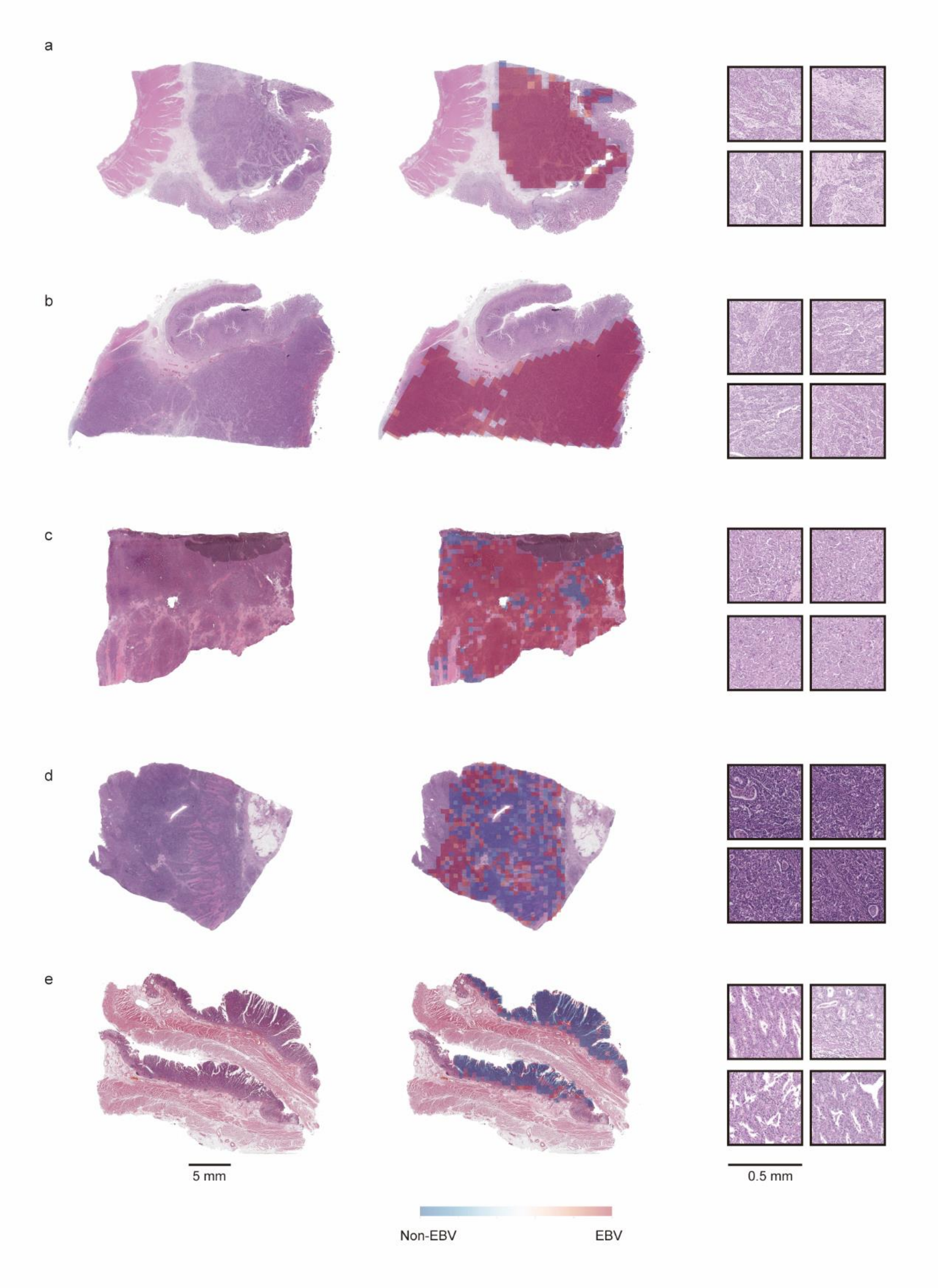

Supplementary Fig. 3. **Unsuccessful cases predicted by EBVNet. a-c** Histological image (left column) of patients with EBVnGC in **a-c** were from MultiCenter-STAD, MultiCenter-STAD and TCGA-STAD respectively. The heatmaps overlapped on these three WSIs (middle column) showed that tumor tiles were mainly predicted as EBVaGC with a high score (reddish color). Tiles with a high score were mainly localized in areas

of medullary histology, poor differentiation, and tumor with vacuolar nucleus or recognizable nucleolus (right column, tiles at 10× magnification). **d-e** Histological image (left column) of patients with EBVaGC in **d-e** were from Internal-STAD and TCGA-STAD respectively. The heatmaps overlapped on these three WSIs (middle column) showed that tumor tiles were mainly predicted as EBVnGC with a low EBV score (bluish color). All results could be reproduced stably by EBVNet. Tiles with a low score were more likely localized in areas of adenoid differentiation, mucinous differentiation, and signet-ring cell differentiation (right column, tiles at 10× magnification). EBV, Epstein-Barr Virus; EBVaGC, Epstein-Barr Virus associated gastric cancer; EBVnGC, Epstein-Barr Virus negative gastric cancer.

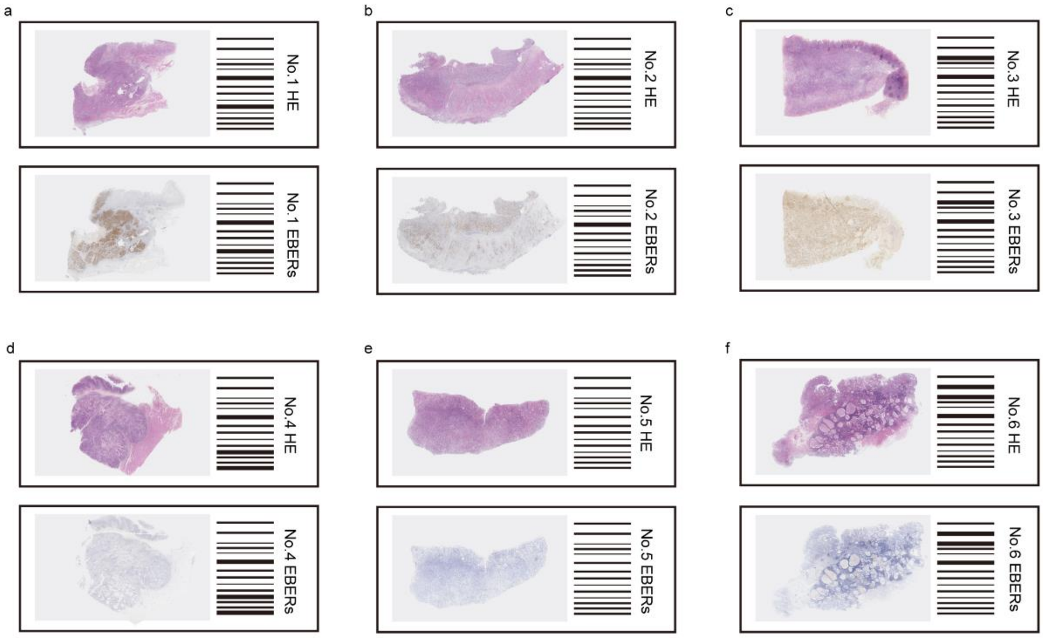

Supplementary Fig. 4. **Determination of EBV status using ISH targeting EBERs in histopathologic samples. a-c** H&E-stained and the corresponding EBERs slides from three patients with EBVaGC. **d-f** H&E and the paired EBERs slides from three patients with EBVnGC. ISH, in situ hybridization; EBERs, Epstein-Barr Virus encoded RNAs; EBVaGC, Epstein-Barr Virus associated gastric cancer; EBVnGC, Epstein-Barr Virus negative gastric cancer.

Supplementary Tables

Supplementary Table 1. Statistics of study participants

Clinicopathological features	Internal-STAD	Multicenter-STAD	TCGA-STAD	*P*
Patients	727	417	239	
Slides	1006	417	258	
EBV status				<0.001
EBV positive slides	203 (20.2%)	98 (23.5%)	24 (9.3%)	
EBV negative slides	803 (79.8%)	319 (76.5%)	234 (90.7%)	
Age (Mean age, ±SD)	59.197±11.367	58.928±12.191	65.583±10.598	<0.001
Gender				0.066
Male	729 (72.5%)	293 (70.3%)	168 (65.1%)	
Female	277 (27.5%)	124 (29.7%)	90 (34.9%)	
Differentiation				<0.001
Poor differentiation	672 (66.8%)	272 (65.2%)	121 (46.9%)	
Middle and well differentiation	334 (33.2%)	145 (34.8%)	137 (53.1%)	
Lauren type				<0.001
Intestinal type	404 (40.2%)	246 (59.0%)	178 (69.0%)	
Diffused type	308 (30.6%)	103 (24.7%)	53 (20.5%)	
Mixed type	294 (29.2%)	68 (16.3%)	27 (10.5%)	

Note: Internal-STAD, the internal dataset; MultiCenter-STAD, external dataset from multiple medical centers; TCGA-STAD, external dataset from The Cancer Genome Atlas. The baseline data of study participants from different datasets were compared by variance analysis or Chi-square test. The *P* value was evaluated from a two-sided test. Adjustments were made for multiple comparisons.

Supplementary Table 2. Diagnostic performances of tumor detector on the external datasets

Datasets	MultiCenter-STAD				TCGA-STAD			
Fold	Sensitivity	Precision	Specificity	AUROC	Sensitivity	Precision	Specificity	AUROC
Fold 1	0.959	0.602	0.541	0.848	0.941	0.679	0.537	0.849
Fold 2	0.974	0.604	0.536	0.873	0.976	0.676	0.514	0.875
Fold 3	0.976	0.567	0.460	0.842	0.960	0.648	0.458	0.841
Fold 4	0.992	0.556	0.426	0.895	0.969	0.633	0.415	0.846
Fold 5	0.918	0.634	0.615	0.850	0.881	0.692	0.592	0.832
Averaged	0.964	0.593	0.515	0.862	0.945	0.503	0.666	0.848

Note: MultiCenter-STAD, external dataset from multiple medical centers; TCGA-STAD, external dataset from The Cancer Genome Atlas; AUROC, area under the receiver operating curve.

Supplementary Table 3. Comparison of different models predicting EBV status on Internal-STAD

Model	Sensitivity	Specificity	NPV	PPV	AUROC	Parameters	Flops
ResNet50	0.857	0.903	0.962	0.701	0.969	23.5M	4.12G
DenseNet121	0.892	0.867	0.970	0.643	0.963	7.0M	2.88G
ResNet18	0.906	0.883	0.974	0.667	0.962	11.2M	1.82G
VGGNet16	0.926	0.920	0.980	0.754	0.973	134.3M	15.53G
SE_ResNet50	0.995	0.517	0.998	0.357	0.963	27.1M	3.89G
EfficientNetB0	0.882	0.907	0.968	0.710	0.968	5.3M	0.39G
EfficientNetB1	0.882	0.893	0.968	0.685	0.968	7.8M	0.70G

Note: Flops were calculated using https://github.com/Swall0w/torchstat. NPV, negative predictive value; PPV, positive predictive value; AUROC, area under the receiver operating curve.

Supplementary Table 4. Performance of five-fold cross validation and test on Internal-STAD

Folds	Sensitivity	Specificity	NPV	PPV	AUROC
Fold 1	0.900<br>(0.76, 0.97)	0.868<br>(0.81, 0.92)	0.972<br>(0.93, 0.99)	0.632<br>(0.49, 0.76)	0.954<br>(0.92, 0.98)
Fold 2	0.878<br>(0.74, 0.96)	0.876<br>(0.81, 0.92)	0.966<br>(0.92, 0.99)	0.643<br>(0.50, 0.77)	0.976<br>(0.96, 1.00)
Fold 3	0.927<br>(0.80, 0.98)	0.913<br>(0.86, 0.95)	0.980<br>(0.94, 1.00)	0.731<br>(0.59, 0.84)	0.981<br>(0.97, 1.00)
Fold 4	0.732<br>(0.57, 0.86)	0.963<br>(0.92, 0.99)	0.934<br>(0.88, 0.97)	0.833<br>(0.67, 0.94)	0.974<br>(0.96, 0.99)
Fold 5	0.850<br>(0.70, 0.94)	0.894<br>(0.84, 0.94)	0.960<br>(0.91, 0.99)	0.667<br>(0.52, 0.79)	0.961<br>(0.94, 0.99)
Averaged	0.857	0.903	0.962	0.701	0.969

Note: 95% confidence intervals are included in brackets. Internal-STAD, the internal dataset; NPV, negative predictive value; PPV, positive predictive value; AUROC, area under the receiver operating curve.

Supplementary Table 5. Diagnostic performances of randomly including slides with 9% proportion of EBVaGC on the MultiCenter-STAD

Random time	AUROC
Random1	0.936 (0.89, 0.98)
Random2	0.954 (0.93, 0.98)
Random3	0.927 (0.88, 0.98)
Random4	0.922 (0.88, 0.97)
Random5	0.954 (0.93, 0.98)
Random6	0.948 (0.92, 0.98)
Random7	0.955 (0.94, 0.98)
Random8	0.940 (0.91, 0.97)
Random9	0.932 (0.90, 0.97)
Random10	0.957 (0.93, 0.98)
Averaged	0.943

Note: MultiCenter-STAD, external dataset from multiple medical centers; EBVaGC, EBV associated gastric cancer; AUROC, area under the receiver operating curve.

Supplementary Table 6. Diagnostic performances of EBVNet using different random seeds on different datasets

Internal-STAD					
Seed	Sensitivity	Specificity	NPV	PPV	AUROC
Seed 0	0.857	0.903	0.962	0.701	0.969
Seed 1	0.936	0.858	0.982	0.638	0.965
Seed 2	0.980	0.699	0.994	0.459	0.974
Seed 3	0.990	0.655	0.996	0.422	0.971
Seed 4	0.907	0.886	0.975	0.681	0.967
Averaged	0.934	0.800	0.982	0.580	0.969
Multicenter-STAD					
Seed	Sensitivity	Specificity	NPV	PPV	AUROC
Seed 0	0.882	0.838	0.962	0.646	0.938
Seed 1	0.916	0.803	0.970	0.592	0.934
Seed 2	0.988	0.500	0.993	0.381	0.930
Seed 3	0.990	0.438	0.993	0.352	0.937
Seed 4	0.861	0.830	0.955	0.634	0.934
Averaged	0.927	0.682	0.975	0.521	0.935
TCGA-STAD					
Seed	Sensitivity	Specificity	NPV	PPV	AUROC
Seed 0	0.658	0.878	0.963	0.377	0.882
Seed 1	0.783	0.809	0.975	0.308	0.891
Seed 2	0.967	0.570	0.994	0.189	0.893
Seed 3	0.967	0.562	0.994	0.186	0.897
Seed 4	0.958	0.538	0.993	0.184	0.898
Averaged	0.867	0.671	0.984	0.249	0.892

Note: Internal-STAD, the internal dataset; MultiCenter-STAD, external dataset from multiple medical centers; TCGA-STAD, external dataset from The Cancer Genome Atlas; NPV, negative predictive value; PPV, positive predictive value; AUROC, area under the receiver operating curve. Mean, mean value; SD, standard deviation.

Supplementary Table 7. Diagnostic performances of different human-machine fusion strategies on external datasets

External datasets	Pathologists	Fusion strategies (AUROC)			
		1/uncertainty (ours)	1-uncertainty	'And'	'Or'
MultiCenter-STAD	Junior1	0.945 (0.92, 0.97)	0.937 (0.91, 0.96)	0.797 (0.76, 0.84)	0.849 (0.81, 0.88)
	Junior2	0.951 (0.93, 0.97)	0.947 (0.93, 0.97)	0.798 (0.76, 0.84)	0.847 (0.81, 0.88)
	Senior1	0.960 (0.94, 0.98)	0.944 (0.92, 0.97)	0.814 (0.77, 0.85)	0.861 (0.82, 0.89)
	Senior2	0.960 (0.94, 0.98)	0.942 (0.92, 0.97)	0.783 (0.74, 0.82)	0.864 (0.83, 0.90)
	Expert1	0.960 (0.94, 0.98)	0.948 (0.93, 0.97)	0.819 (0.78, 0.86)	0.866 (0.83, 0.90)
	Expert2	0.969 (0.95, 0.98)	0.951 (0.93, 0.98)	0.816 (0.78, 0.85)	0.864 (0.83, 0.90)
TCGA-STAD	Junior1	0.915 (0.87, 0.97)	0.912 (0.87, 0.96)	0.674 (0.61, 0.73)	0.778 (0.72, 0.83)
	Junior2	0.916 (0.87, 0.97)	0.920 (0.88, 0.97)	0.668 (0.61, 0.73)	0.774 (0.73, 0.82)
	Senior1	0.925 (0.89, 0.97)	0.851 (0.77, 0.94)	0.664 (0.60, 0.72)	0.823 (0.77, 0.87)
	Senior2	0.928 (0.89, 0.96)	0.931 (0.90, 0.96)	0.693 (0.63, 0.75)	0.795 (0.74, 0.84)
	Expert1	0.931 (0.89, 0.98)	0.908 (0.85, 0.97)	0.724 (0.67, 0.78)	0.821 (0.77, 0.87)
	Expert2	0.939 (0.90, 0.98)	0.932 (0.89, 0.97)	0.724 (0.67, 0.78)	0.825 (0.77, 0.87)

Note: MultiCenter-STAD, external dataset from multiple medical centers; TCGA-STAD, external dataset from The Cancer Genome Atlas; AUROC, area under the receiver operating curve. Junior 1, Junior pathologist 1; Junior 2, Junior pathologist 2; Senior 1, Senior pathologist 1; Senior 2, Senior pathologist 2; Expert 1, Expert pathologist 1; Expert 2, Expert pathologist 2.

Supplementary Table 8. Number of morphological features on different datasets

Morphological features		Internal-STAD (n=1006)	Multicenter-STAD (n=417)	TCGA-STAD (n=258)
Tertiary lymphoid structure	Presence	698 (69.3%)	253 (60.7%)	133 (51.6%)
	Absence	308 (30.7%)	164 (39.3%)	125 (48.4%)
Medullary histology	Presence	96 (9.5%)	77 (18.5%)	15 (5.8%)
	Absence	910 (90.5%)	340 (81.5%)	243 (94.2%)
Mucinous differentiation	Presence	187 (18.6%)	57 (13.7%)	50 (19.4%)
	Absence	819 (81.4%)	360 (86.3%)	208 (80.6%)
Adenoid differentiation	Presence	602 (59.8%)	240 (57.6%)	165 (64.0%)
	Absence	404 (40.2%)	177 (42.4%)	93 (36.0%)
Papillary differentiation	Presence	212 (21.1%)	63 (15.1%)	83 (32.2%)
	Absence	794 (78.9%)	354 (84.9%)	175 (67.8%)
Signet-ring cell	Presence	192 (19.1%)	101 (24.2%)	37 (14.3%)
	Absence	814 (80.9%)	316 (75.8%)	221 (85.7%)
Poor differentiation	Presence	672 (66.8%)	272 (65.2%)	121 (46.9%)
	Absence	334 (33.2%)	145 (34.8%)	137 (53.1%)
Vacuolar nucleus or recognizable nucleolus	Presence	480 (47.7%)	216 (51.8%)	120 (46.5%)
	Absence	526 (52.3%)	201 (48.2%)	138 (53.5%)

Note: Percentages are included in brackets. Internal-STAD, the internal dataset; MultiCenter-STAD, an external dataset from multiple medical centers; TCGA-STAD, an external dataset from The Cancer Genome Atlas.

Supplementary Table 9. The comparison of EBVNet misdiagnosed and correctly diagnosed cases on MultiCenter-STAD dataset

Clinicopathological features		EBVaGC			EBVnGC		
		False negative (n=3)	True positive (n=95)	*P*	False positive (n=77)	True negative (n=242)	*P*
Mean age		60.33±13.013	57.27±12.308	0.673	59.64±12.574	59.33±12.032	0.850
Gender	Male	3 (100.0%)	84 (88.4%)	1.000	41 (53.2%)	165 (68.2%)	0.017
	Female	0 (0.0%)	11 (11.6%)		36 (46.8%)	77 (31.8%)	
Lauren type	Intestinal	1 (33.3%)	69 (72.6%)	0.196	37 (48.1%)	139 (57.4%)	0.149
	Others*	2 (66.7%)	26 (27.4%)		40 (51.9%)	103 (42.6%)	
Tertiary lymphoid structure	Presence	3 (100.0%)	66 (69.5%)	0.553	48 (62.3%)	136 (56.2%)	0.342
	Absence	0 (0.0%)	29 (30.5%)		29 (37.7%)	106 (43.8%)	
Medullary histology	Presence	0 (0.0%)	63 (66.3%)	0.080	13 (16.9%)	1 (0.4%)	<0.001
	Absence	3 (100.0%)	32 (33.7%)		64 (83.1%)	241 (99.6%)	
Mucinous differentiation	Presence	0 (0.0%)	0 (0.0%)	NA	6 (7.8%)	51 (21.1%)	0.008
	Absence	3 (100.0%)	95 (100.0%)		71 (92.2%)	191 (78.9%)	
Adenoid differentiation	Presence	1 (33.3%)	46 (48.4%)	1.000	31 (40.3%)	162 (66.9%)	<0.001
	Absence	2 (66.7%)	49 (51.6%)		46 (59.7%)	80 (33.1%)	
Papillary differentiation	Presence	0 (0.0%)	1 (1.0%)	1.000	7 (9.1%)	55 (22.7%)	0.008
	Absence	3 (100.0%)	94 (99.0%)		70 (90.9%)	187 (77.3%)	
Signet-ring cell	Presence	1 (33.3%)	3 (3.2%)	0.119	22 (28.6%)	75 (31.0%)	0.688
	Absence	2 (66.7%)	92 (96.8%)		55 (71.4%)	167 (69.0%)	
Poor differentiation	Presence	3 (100.0%)	86 (90.5%)	1.000	65 (84.4%)	118 (48.8%)	<0.001
	Absence	0 (0.0%)	9 (9.5%)		12 (15.6%)	124 (51.2%)	
Vacuolar nucleus or recognizable nucleolus	Presence	1 (33.3%)	81 (85.3%)	0.068	43 (55.8%)	91 (37.6%)	0.005
	Absence	2 (66.7%)	14 (14.7%)		34 (44.2%)	151 (62.4%)	

Note: *Others indicated diffused type and mixed type. 95% confidence intervals are included in brackets. MultiCenter-STAD, external dataset from multiple medical centers; EBVaGC, EBV associated gastric cancer; EBVnGC, EBV negative gastric cancer. The features of misdiagnosed cases were compared with those of correctly diagnosed cases with the Chi-square test or *t* test. The *P* value was evaluated from a two-sided test.

Supplementary Table 10. The comparison of EBVNet misdiagnosed and correctly diagnosed cases on TCGA-STAD dataset

Clinicopathological features		EBVaGC			EBVnGC		
		False negative cases (n=5)	True positive cases (n=19)	*P*	False positive cases (n=39)	True negative cases (n=195)	*P*
Mean age		69.20±8.289	59.63±10.172	0.066	66.59±10.500	65.86±10.604	0.697
Gender	Male	5 (100.0%)	15 (78.9%)	0.544	16 (41.0%)	132 (67.7%)	0.002
	Female	0 (0.0%)	4 (21.1%)		23 (59.0%)	63 (32.3%)	
Lauren type	Intestinal	3 (60.0%)	13 (68.4%)	1.000	28 (71.8%)	134 (68.7%)	0.704
	Others	2 (40.0%)	6 (31.6%)		11 (28.2%)	61 (31.3%)	
Tertiary lymphoid structure	Presence	1 (20.0%)	11 (57.9%)	0.317	23 (59.0%)	98 (50.3%)	0.320
	Absence	4 (80.0%)	8 (42.1%)		16 (41.0%)	97 (49.7%)	
Medullary histology	Presence	0 (0.0%)	6 (31.6%)	0.280	7 (17.9%)	2 (1.0%)	<0.001
	Absence	5 (100.0%)	13 (68.4%)		32 (82.1%)	193 (99.0%)	
Mucinous differentiation	Presence	0 (0.0%)	0 (0%)	NA	9 (23.1%)	41 (21.0%)	0.775
	Absence	5 (100.0%)	19 (100%)		30 (76.9%)	154 (79.0%)	
Adenoid differentiation	Presence	3 (60.0%)	8 (42.1%)	0.630	20 (51.3%)	134 (68.7%)	0.036
	Absence	2 (40.0%)	11 (57.9%)		19 (48.7%)	61 (31.3%)	
Papillary differentiation	Presence	2 (40.0%)	2 (10.5%)	0.179	2 (5.1%)	77 (39.5%)	<0.001
	Absence	3 (60.0%)	17 (89.5%)		37 (94.9%)	118 (60.5%)	
Signet-ring cell	Presence	1 (20.0%)	1 (5.3%)	0.380	4 (10.3%)	31 (15.9%)	0.367
	Absence	4 (80.0%)	18 (94.7%)		35 (89.7%)	164 (84.1%)	
Poor differentiation	Presence	2 (40.0%)	16 (84.2%)	0.078	26 (66.7%)	77 (39.5%)	0.002
	Absence	3 (60.0%)	3 (15.8%)		13 (33.3%)	118 (60.5%)	
Vacuolar nucleus or recognizable nucleolus	Presence	2 (40.0%)	14 (73.7%)	0.289	28 (71.8%)	76 (39.0%)	<0.001
	Absence	3 (60.0%)	5 (26.3%)		11 (28.2%)	119 (61.0%)	

Note: *Others indicated diffused type and mixed type. TCGA-STAD, external dataset from The Cancer Genome Atlas; EBVaGC, EBV associated gastric cancer; EBVnGC, EBV negative gastric cancer. The features of misdiagnosed cases were compared with those of correctly diagnosed cases with the Chi-square test or *t* test. The *P* value was evaluated from a two-sided test.

Supplementary References

1. Vahadane A, *et al.* Structure-Preserving Color Normalization and Sparse Stain Separation for Histological Images. *IEEE Trans Med Imaging* **35**, 1962-1971 (2016).

2. Zhou W, *et al.* Ensembled deep learning model outperforms human experts in diagnosing biliary atresia from sonographic gallbladder images. *Nat Commun* **12**, 1259 (2021).
